# Optimizing Phakic Intraocular Lens Sizing: Navigating the Complexities of Nomograms

**DOI:** 10.22336/rjo.2026.50

**Published:** 2026

**Authors:** Shreesha Kumar Kodavoor, Sandip Dattatray Patil, Parth Palan, Dandapani Ramamurthy

**Affiliations:** 1 Department of Cornea, Cataract, Refractive Surgery, The Eye Foundation, Coimbatore, Tamil Nadu, India; 2 Department of Cataract, Refractive Surgery, The Eye Foundation, Tirunelveli, India; 3 The Eye Foundation Group of Hospitals, India

**Keywords:** phakic intraocular lens, white-to-white diameter, sulcus-to-sulcus diameter, sizing nomogram, vault prediction formulas, pIOL = Phakic intraocular lens, ICL = Implantable Collamer Lens, IPCL = Implantable Phakic Contact Lens, IOP = Intraocular pressure, ACD = Anterior Chamber Depth, ATA =Angle to Angle distance, WTW = White to White, STS = Sulcus to Sulcus, LV = Lens vault, CLR = crystalline lens rise, UBM = ultrasound biomicroscopy, ASOCT = anterior segment optical coherence tomography, CBID = ciliary body inner diameter

## Abstract

Phakic intraocular lenses (pIOLs) provide refractive correction while maintaining the natural lens and the cornea’s asphericity. Accurate measurement of anterior segment parameters is essential for optimal pIOL selection and sizing. Various instruments, such as optical biometers, topographers, anterior segment OCT, and UBM, allow for a comprehensive evaluation. Nomograms and formulas further fine-tune pIOL sizing by considering factors like white-to-white diameter, anterior chamber depth, corneal curvature, and sulcus-to-sulcus diameter. This review article examines current technologies and strategies for optimizing pIOL nomograms. It highlights the key factors that influence the development of nomograms. Furthermore, the article examines clinical studies comparing different nomogram outcomes and challenges associated with them. Postoperative vault assessment ensures sufficient clearance between the pIOL and crystalline lens, reducing the risk of complications. Integrating these technologies and algorithms improves the accuracy and safety of pIOL implantation, enhancing visual outcomes for refractive surgery patients.

## Introduction

Over the last two decades, the use of phakic intraocular lens (pIOL) implantation has surged in popularity as an efficient refractive solution for correcting moderate to high ametropia in candidates who are not suitable for laser-based keratorefractive surgeries. The Visian Implantable Collamer Lens (ICL, Staar Surgical) offers a correction range from +10D to -20D, with cylindrical correction up to 6D, and is available in four sizes: 12.1 mm, 12.6 mm, 13.2 mm, and 13.7 mm. Other phakic intraocular lenses (pIOLs) manufactured in India include the Implantable Phakic Contact Lens (IPCL; Care Group, Vadodara, India), which provides a correction range from +15D to -30D with cylindrical power up to 10D and is available in sizes with an incremental increase of 0.25 mm from 11 to 14 mm. Other phakic IOLs available in the Indian market include the Refractive Implantable Lens (RIL; Appasamy Associates, Chennai, India) and the Eyecryl phakic IOL (Biotech Vision Care, Ahmedabad, India).

The V4c Visian Implantable Collamer Lens, designed with a 360-micron central hole, can effectively correct moderate to high myopia with few complications, including anterior subcapsular cataract, increased intraocular pressure (IOP), endothelial cell loss, pigment dispersion, pupillary block, and glaucoma. Additionally, the pIOL procedure is reversible, improves visual acuity, and maintains excellent refractive stability while preserving the eye’s natural ability to accommodate [1].

The IPCL V2.0, made from reinforced hybrid hydrophilic material, provides an extensive range of vision correction and features a 380-micron central hole for optimal aqueous flow. It also offers a presbyopia correction option of up to 4D [2].

## Anterior segment parameters

The sizing of phakic intraocular lenses (pIOLs) is crucial in pIOL implantation as it significantly impacts vault height and subsequent postoperative outcomes. A vault height that is too low increases the risk of contact between the implanted lens and the crystalline lens, potentially leading to cataracts. Conversely, a vault height that is too high can result in angle-closure glaucoma, endothelial cell damage, and pigment dispersion. A postoperative vault height of 250–750 µm is widely regarded as a safe target [3]. Several parameters must be considered for accurate sizing, including preoperative measurements of Anterior Chamber Depth (ACD), Angle to Angle distance (ATA), White to White (WTW), and Sulcus to Sulcus (STS).

### Anterior Chamber Depth (ACD)

Anterior chamber depth (ACD) is a well-established biometric parameter of the anterior segment. Anatomically, it denotes the distance between the corneal endothelium and the anterior capsule of the crystalline lens [4]. An anterior chamber depth (ACD) of 3.00 mm or greater is necessary for implantation; a shallower ACD can result in angle crowding and an increased risk of glaucoma [5].

### Angle to Angle distance (ATA)

The angle-to-angle diameter measures the horizontal span of the anterior chamber, extending from one iridocorneal angle to the opposite angle. This measurement provides crucial information about the size and configuration of the anterior chamber, which is essential for evaluating the risk of angle-closure glaucoma, assessing anterior chamber stability, and planning anterior segment surgeries. The ATA distance is automatically obtained by drawing a line between the iris roots on opposite sides using anterior segment optical coherence tomography. Igarashi et al. developed a formula based on ATA distance for a Japanese population, revealing a significant correlation between ICL size, postoperative vault, and ATA [6].

### White to White (WTW)

The white-to-white (WTW) distance represents the visible horizontal corneal diameter, measured between the edges of the corneal limbus. Various techniques can be employed for WTW measurement, such as manual calipers, digital calipers, optical biometry devices (such as the IOLMaster), anterior segment imaging modalities (like AS-OCT, Scheimpflug imaging), and Fourier-based profilometry [7]. Each method has its strengths and weaknesses, with considerations such as accuracy, precision, ease of use, and patient comfort influencing the selection of the approach. Manual calipers were conventionally utilized for WTW measurement but have largely been supplanted by more sophisticated and automated methods that provide enhanced accuracy and reproducibility. These advanced techniques allow for non-invasive determination of corneal diameter.

### Sulcus to Sulcus (STS)

The sulcus-to-sulcus diameter represents the horizontal measurement of the ciliary sulcus, which is the area between the posterior surface of the iris and the anterior surface of the ciliary body [8]. STS measurement can be performed using various techniques, including anterior segment imaging modalities such as anterior segment optical coherence tomography (AS-OCT CASIA2) [9], machines using ultrasound like ArcScan Insight 100 (very high frequency automated digital ultrasound) [10], Vumax II (wide-scanning-field ultrasound biomicroscopy), and HiScan imaging. These imaging methods offer detailed views of the ciliary sulcus, enabling accurate measurement of the sulcus-to-sulcus diameter. This can be particularly challenging in cases of anterior segment irregularities, such as a narrow or irregular ciliary sulcus, or in patients with significant media opacities that hinder clear visualization.

### Correlation Between WTW and Posterior Chamber

While manufacturers have promoted lens sizing through white-to-white (WTW) measurements obtained via manual or automated techniques, along with the consideration of anterior chamber depth measurements to determine pIOL suitability, a study conducted by Liliana Wilmer et al. revealed a positive correlation between white-to-white measurements and anterior chamber diameter, but no correlation between white-to-white measurements and ciliary sulcus diameter [11,12]. Additionally, M. Pop et al., in their study on predicting sulcus size using ocular measurements, concluded that the traditional estimation of sulcus size through limbal measurement alone is insufficient, as limbus size alone cannot reliably predict sulcus size [13].

Reinstein et al. demonstrated that predicting sulcus diameter using white-to-white measurements shows weak correlations (r² = 0.32). They suggested that direct measurement of the sulcus diameter would enhance the safety of sizing for both anterior and posterior chamber phakic IOLs [14]. Including sulcus diameter in the ICL sizing formula significantly improved the predictability of postoperative vault height compared to the traditional formula based solely on white-to-white measurements [15].

## Anterior segment measurements

### Direct Measurement

Both manual and digital calipers are commonly used to measure the white-to-white (WTW) diameter in ophthalmic practice. Traditional manual calipers usually have increments of 0.2 mm, while digital calipers provide readings in increments of 0.1 mm. Both types can offer accurate WTW measurements when used correctly (**Fig. 1**). However, digital calipers may provide slightly higher accuracy due to their digital readout and finer measurement resolution. Baumeister compared manual and automated methods for determining horizontal corneal diameter and found that automated measurements of the WTW distance are more precise than those obtained using manual methods [16].

**Fig. 1:**
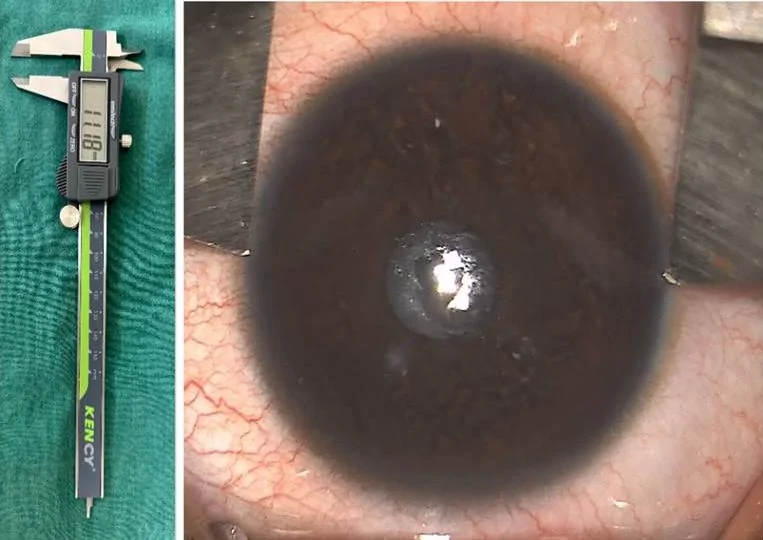
Digital caliper and its use for white-to-white measurement

### Optical Biometer

Optical biometry also provides white-to-white measurements, which can be cross-referenced with manual measurements or those obtained from other devices for verification. Devices like the IOLMaster and Lenstar LS 900 are suitable for measuring WTW and angle-to-angle distances in clinical practice (**Fig. 2,3**).

**Fig. 2:**
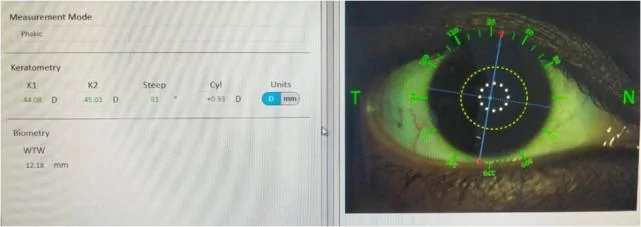
Optical biometer screen showing white-to-white diameter

**Fig. 3:**
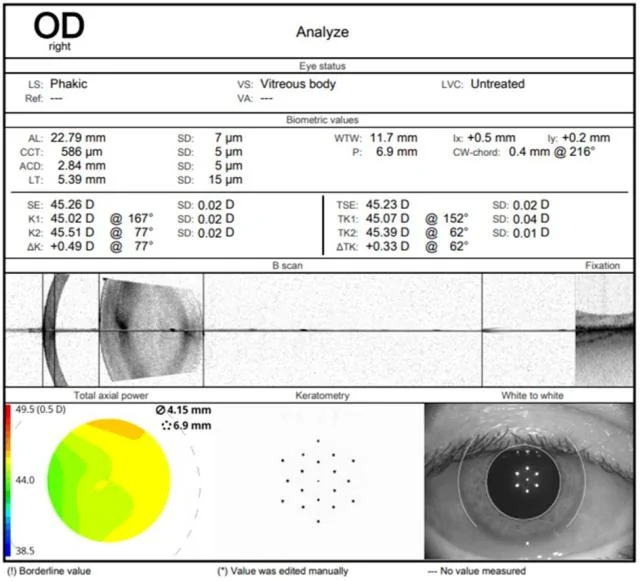
IOL MASTER 700 biometer screen showing white-to-white diameter

### Corneal Topography

Topographers, whether slit scanning (Orbscan II), Placido-based (Sirius), or Scheimpflug (Pentacam), enable the calculation of WTW values using both manual and automated modes [17]. Manual measurements were found to be less accurate than automatic modes, possibly due to the difficulty in visualizing the transition zone from the cornea to the sclera. The accuracy of automated limbus recognition depends on image quality, allowing for precise measurements of white-to-white diameter (WTW), anterior chamber depth (ACD), and keratometry (K).

The STAAR Surgical Nomogram and the Optimized WTW Nomogram use these measurements to recommend ICL sizes. While the Optimized WTW Nomogram is similar to the STAAR Surgical Nomogram, it has a lower tendency to predict oversized ICLs, particularly for larger sizes. A study by Moshirfar et al. found that these nomograms often predicted oversized lenses, resulting in higher-than-expected vaults. Therefore, they may not be ideal for ICL sizing for larger lenses but could be advantageous for patients with a low predicted postoperative vault or smaller lens sizes [18] (**Fig. 4,5**).

**Fig. 4:**
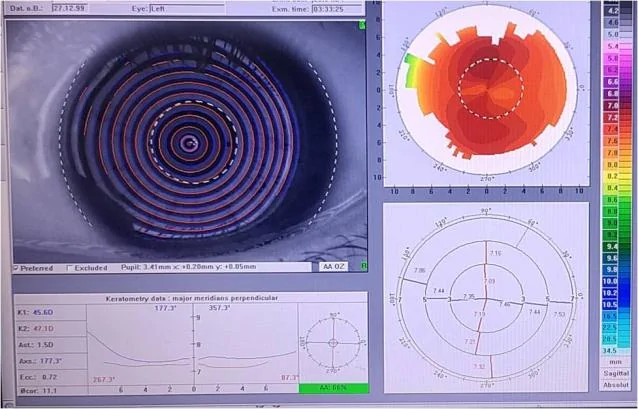
Topolyser image

**Fig. 5:**
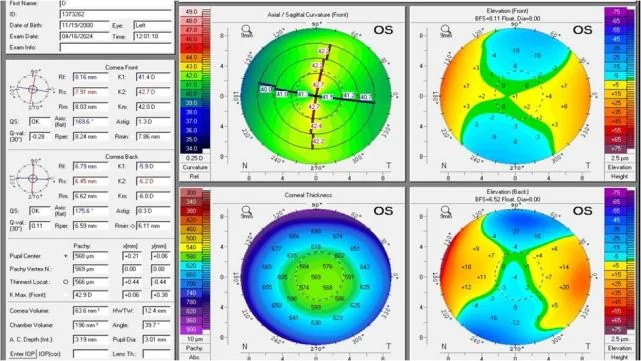
QUAD MAP of PENTACAM with summary indices showing white-to-white measurement

### Anterior Segment OCT

Anterior Segment OCT technology enables the automatic measurement of various anterior segment parameters, such as central corneal thickness (CCT), anterior chamber depth (ACD), angle-to-angle distance (ATA), angle-to-angle width (ACW, the distance between two scleral spurs), lens vault (LV, the vertical distance between a line connecting two scleral spurs and the anterior surface of the crystalline lens), and crystalline lens rise (CLR, the vertical distance between a line connecting two angle recesses and the anterior surface of the crystalline lens). Studies have demonstrated that the anterior segment parameters measured by CASIA-2 exhibit high inter- and intra-examiner reproducibility, except for LV, possibly due to the accommodation effect [19]. AS-OCT appears to be a more practical method, offering nearly equivalent accuracy to the UBM method in clinical settings. It allows multiple automatic measurements in a single capture, while remaining non-contact and comfortable for the patient. Nomograms using ASOCT parameters are Nakamura nomogram (NK V1-V3), KS nomogram (KS V1-V2) and Kim nomogram.

The NK Nomogram focuses on anterior chamber width and crystalline lens rise to calculate the optimal ICL size. Through optimization from NK1 to NK2, they minimized the selection of larger ICL sizes [20], resulting in higher accuracy for predicting vault compared to the STAAR nomogram [21]. Furthermore, the reduction of the horizontal compression coefficient from 1.1, initially calculated from in vitro studies in NK2, may not hold in human eyes. It was subsequently reduced to 0.729, which exhibited less error in predicting postoperative vaults [22].

The KS nomogram also comes in two versions and utilizes WTW and ATA measurements obtained from AS-OCT. The second version (KS V2) was created to address the finding that ICL size affected vault prediction error (postoperative vault - predicted vault) [6].

The KIM nomogram, another nomogram utilizing aqueous depth, anterior chamber angle, and lens thickness obtained from ANTERION ASOCT, demonstrates a strong correlation between predicted vault, ICL size, and the aforementioned parameters [23] (**Fig. 6,7**).

**Fig. 6:**
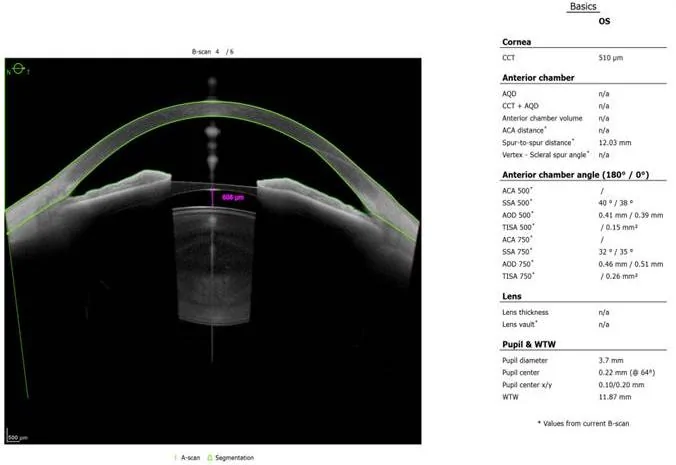
Screen of ASOCT ANTERION showing various parameters like WTW and postoperative vault

**Fig. 7:**
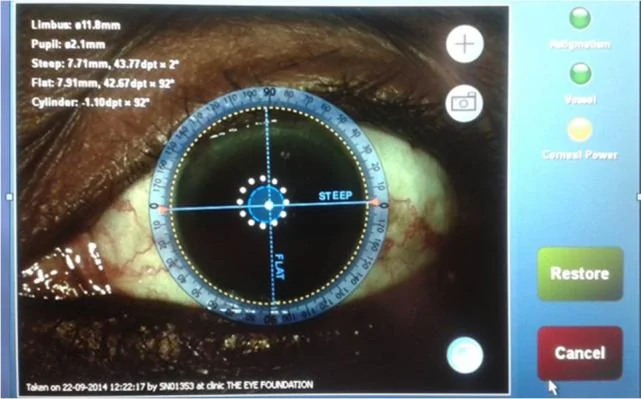
Use of image-guided system VERION for measurements

### Ultrasound

Direct observation of the posterior chamber involves assessing parameters such as the inner diameter of the ciliary body and the zonule-to-zonule distance, in addition to angle parameters. This examination aids in identifying and eliminating iris or ciliary pathologies such as cysts, which were found to occur in approximately 26.87% of cases in a study by Zhen Li et al. [24]. Nomograms based on ultrasound include those developed by Reinstein, Dougherty & Parkhurst, Kojima, and ZZ.

The Reinstein nomogram focuses on parameters such as the ciliary body inner diameter, sulcus-to-sulcus lens rise, scotopic pupil size, ICL power, and size. According to this model, the ciliary body inner diameter was the most predictive factor for postoperative vault height (**Fig. 8**). The calculator provides predicted vault measurements for each available size of ICL, along with lens size recommendations based on the Dougherty, Kojima, and Nakamura formulas. A study conducted by this group comparing their model with other established ICL sizing nomograms found a significantly lower interquartile range of predicted vault height (131 microns), with 94% of eyes falling within 300 microns of the targeted vault height [25].

**Fig. 8:**
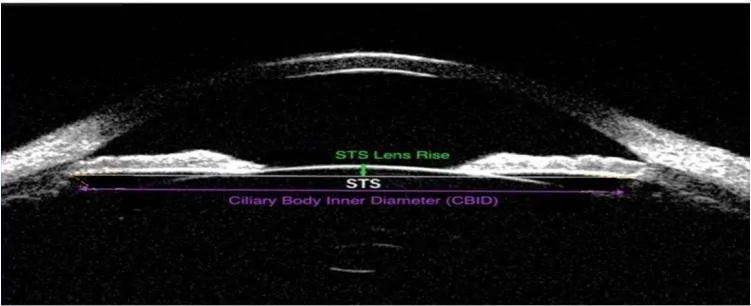
Ultrasound biomicroscopy image of posterior chamber structures and parameters used for various nomograms

Both the Dougherty and Parkhurst nomograms rely on the measurement of sulcus-to-sulcus (STS) distance. However, the Parkhurst nomogram incorporates crystalline lens rise (CLR) alongside STS and has been observed to offer superior predictive accuracy for pIOL size compared to the Dougherty nomogram [18].

Kojima et al. devised a formula utilizing preoperative measurements acquired via UBM, based on parameters such as ACD, STS, and STSL. The distribution of postoperative vaults at the 3-month mark using this formula was compared to the predicted vaults from manufacturer guidelines and Dougherty’s nomograms. It was determined that the Kojima formula exhibited superiority in selecting for moderate-sized vaults [26].

The ZZ nomogram incorporates horizontal and vertical sulcus-to-sulcus (STS) measurements, the expected orientation of the ICL, anterior chamber depth (ACD), axial length (AL), crystalline lens thickness (CLT), effective lens position, and surgery-induced astigmatism to predict both vault height and ICL sizing. Findings indicated that the predicted postoperative vault using the ZZ formula was notably lower than that projected by the FDA nomogram in patients receiving larger TICLs [27]. This discrepancy could potentially elevate the risk of postoperative complications. Based on these results, the advice is to avoid using this nomogram in patients expected to have a low postoperative vault (**Table 1**) [28]. **Table 1**. Individual nomograms based on different devices with the parameters for pIOL sizing

**Table 1:** Individual nomograms based on different devices with the parameters for pIOL sizing

Device	Nomogram	Parameter
Topographer	STAAR Surgical	WTW, ACD
	Optimized WTW	WTW, ACD
ASOCT	NK nomogram	ACW, CLR
	KS nomogram	ICL size, ATA distance
	KIM nomogram	AQD, ACA distance, LT
UBM	Dougherty	STS
	Parkhurst	STS, CLR
	Kojima	ACD, STS, STSL
	ZZ nomogram	Horizontal/vertical STS, ACD, AL, CLT, ELP, SIA
	Reinstein	CBID, STSL, Scotopic pupil size

*ACA = anterior chamber angle, ACD = anterior chamber depth, ACW = anterior chamber width, AL = axial length, AQD = aqueous depth, ATA = angle-to-angle distance, CBID = ciliary body inner diameter, CLR = crystalline lens rise, CLT = crystalline lens thickness, STS = sulcus-to-sulcus, STSL = sulcus-to-sulcus lens rise, WTW = white-to-white.*

## Repeatability

Before implementing any nomograms in clinical practice, it is crucial to understand repeatability and agreement across devices. In a study by Yokoyama et al. [29], the mean coefficient of variation was found to be 0.62% ± 0.20% for intra-examiner measurements and 3.4% ± 2.4% for inter-examiner measurements of sulcus-to-sulcus diameter using wide-scanning-field ultrasound biomicroscopy (UBM) (Vumax II). These findings suggest that the repeatability of direct sulcus-to-sulcus measurement is sufficiently high for clinical use.

The Artemis 2 and the Visante OCT systems offer comparable and consistent measurements of the anterior chamber depth (ACD), central corneal thickness (CCT), and angle-to-angle distance (ATA), making them interchangeable for these applications [30]. On the other hand, the Arcscan Insight 100 is a very high-frequency ultrasound device (50 MHz) that produces higher resolution images of the posterior chamber, which might be challenging to capture with anterior segment optical coherence tomography (AS-OCT). Its transducer is robotically controlled with autocentration technology, minimizing measurement inconsistencies that can occur with operator-held UBM probes.

## Discussion

Clinicians have access to various nomograms for precise pIOL sizing, depending on the imaging tools and preoperative measurements available. **Table 1** serves as a useful reference for clinicians when selecting the appropriate nomograms [28]. Achieving proper sizing is crucial in pIOL implantation, as it ensures an optimal postoperative vault, thereby reducing the risk of complications such as cataracts and glaucoma. Studies suggest that postoperative vault height usually decreases within the first 3–6 months after surgery and stabilizes within 6–12 months [31].

When using AS-OCT or UBM to measure anterior segment parameters and apply respective nomograms, it is essential to acknowledge the limitations of AS-OCT in detecting more posterior measurements and structures such as the ciliary body, sulcus-to-sulcus distance (STS), and scleral spur. In contrast, UBM utilizes very high-frequency ultrasound (ranging from 35 to 100 MHz) for these measurements, which may be subject to operator error when holding the transducer [32]. Additionally, these nomograms are derived from parameters measured with specific devices, and there may be poor correlation between different devices for the same measurement [33]. Therefore, averaging out the readings may be advisable for optimal sizing. Moreover, most of these nomograms are developed using samples from Asian demographics. Hence, it is prudent to consider race when utilizing these nomograms. It is important to recognize that the ciliary sulcus is a dynamic area, influenced by factors such as accommodation and changes in pupil size under varying light conditions [34]. Measuring the white-to-white diameter with a digital caliper is a widely practiced indirect method for estimating the internal ciliary sulcus dimensions. When combined with an optimized WTW nomogram, this approach serves as a reliable and well-established protocol for determining the appropriate size of phakic IOL. The weak correlation between white-to-white (WTW) and sulcus-to-sulcus (STS) diameters has led to unexpected postoperative phakic IOL vault outcomes, sparking greater interest in the direct visualization and measurement of the ciliary sulcus using very high-frequency ultrasound. This has prompted the development of new nomograms based on sulcus parameters, which are yielding promising results. However, challenges such as interobserver variability and poor reproducibility continue to limit their broader adoption in clinical practice.

## Conclusion

In conclusion, phakic IOL sizing and its influence on vault height have become crucial factors for clinicians when evaluating implantation suitability and the risk of postoperative complications. Nomograms play a vital role in determining the optimal size by using various preoperative measurements. This highlights the ongoing effort to improve ICL sizing protocols, aiming for greater precision and better outcomes in clinical practice.
